# Combining texture features of whole slide images improves prognostic prediction of recurrence-free survival for cutaneous melanoma patients

**DOI:** 10.1186/s12957-020-01909-5

**Published:** 2020-06-16

**Authors:** Yanbin Peng, Yunfeng Chu, Zhong Chen, Wen Zhou, Shengxiang Wan, Yingfeng Xiao, Youlong Zhang, Jialu Li

**Affiliations:** 1 Department of Microsurgery, Peking University Shenzhen Hospital, Futian District, Shenzhen, 518036 China; 2 Department of Biostatistics, HuaJia Biomedical Intelligence, Shenzhen Overseas Chinese High-Tech Venture Park, Nanshan District, Shenzhen, 518057 China

**Keywords:** Cutaneous melanoma, Recurrence-free survival, Whole slide image, Computer-aided image processing

## Abstract

**Background:**

Accurate prediction of recurrence-free survival (RFS) is important for the prognosis of cutaneous melanoma patients. The image-based pathological examination remains as the gold standard for diagnosis. It is of clinical interest to account for computer-aided processing of pathology image when performing prognostic analysis.

**Methods:**

We enrolled in this study a total of 152 patients from TCGA-SKCM (The Cancer Genome Atlas Skin Cutaneous Melanoma project) with complete information in recurrence-related survival time, baseline variables (clinicopathologic variables, mutation status of BRAF and NRAS genes), gene expression data, and whole slide image (WSI) features. We preprocessed WSI to segment global or nucleus areas, and extracted 3 types of texture features from each region. We performed cross validation and used multiple evaluation metrics including C-index and time-dependent AUC to determine the best model of predicting recurrence events. We further performed differential gene expression analysis between the higher and lower-risk groups within AJCC pathologic tumor stage III patients to explore the underlying molecular mechanisms driving risk stratification.

**Results:**

The model combining baseline variables and WSI features had the best performance among models with any other types of data integration. The prognostic risk score generated by this model could provide a higher-resolution risk stratification within pathologically defined subgroups. We found the selected image features captured important immune-related variations, such as the aberration of expression in T cell activation and proliferation gene sets, and therefore contributed to the improved prediction.

**Conclusions:**

Our study provided a prognostic model based on the combination of baseline variables and computer-processed WSI features. This model provided more accurate prediction than models based on other types of data combination in recurrence-free survival analysis.

**Trial registration:**

This study was based on public open data from TCGA and hence the study objects were retrospectively registered.

## Background

Melanoma is a type of skin cancer with a high mortality rate. In 2018, 287,732 new cases and 60,712 deaths of melanoma were registered worldwide [1]. Cutaneous melanoma, which accounts for over 90% of melanoma cases, remains one of the most aggressive forms of skin cancer and shows an increasing incidence and mortality rate globally [2, 3]. Improving prognosis of cutaneous melanoma patients has important implications for a better management of the disease. Routine prognosis method uses clinicopathologic features including Breslow tumor thickness, ulceration, mitotic index, Clark level, and AJCC (The American Joint Committee on Cancer) pathologic tumor stage [4, 5]. Whether such method can be improved with the addition of WSI or high-throughput sequencing data is under active investigation.

Many previous studies had attempted to develop prognostic models using different types of variables including clinicopathologic, mutation, mRNA, microRNA, and methylation variables. Zhao et al. [6] identified a 25-gene signature that can effectively estimate the level of immune cell infiltration in melanoma, providing a robust biomarker significantly related to survival outcome (disease-specific survival, post-recurrence survival, or overall survival). Multidimensional omics data were also utilized to provide more accurate prediction. Jayawardana et al. [7] developed models to classify 1-year and 4-year survival status based on clinicopathologic, mutation, mRNA, microRNA, protein information, and their different combinations. They identified that models based on the combination of clinicopathologic variables and mRNA expression profile performed the best under a cross-validation framework. Jiang et al. [8] used sparse PCA and partial least squares methods to take whole multidimensional omics profiles into consideration. Their methods showed a significant increase of C-index values of overall survival prediction. However, these studies had not extended to including WSI features, while studies that did use image data were not focused on the prediction of survival outcomes. For example, Lu et al. [9] proposed a diagnostic model based on epidermis segmentation, keratinocytes segmentation, melanocytes detection, and feature construction on whole slide images. This technique achieved a classification accuracy of 90% for skin tissue malignancy. Failmezger et al. [10] analyzed the spatial association between different types of nodes in WSI. They identified that two stromal features (stroma clustering and stromal barrier) had significant coefficients in a Cox model. To our knowledge, few studies proposed pathology image-based prediction models for recurrence-free survival analysis.

As the treatment of cutaneous melanoma has improved over years, especially since the advent of targeted immunotherapy [11, 12], the RFS measurement has gained increasing importance for post-surgery management of melanoma patients. An accurate RFS analysis can decrease the cancer-related death rate by not only personalizing treatment options but also prompting active cancer surveillance at an early stage for specific risk groups. On the other hand, as recurrence events have already been used as an effective endpoint for cancer clinical trials, a higher accuracy of RFS measurements allows precise enrollment of patients that are more likely responsive to new therapies.

Our work hence presented a RFS model based on baseline variables and texture features extracted from WSI. This model provided a higher accuracy in predicting recurrence-free survival than baseline variables-based model or models with other types of data integration. The extracted WSI features contributed to the improved prediction by capturing variations in immune-related gene expressions.

## Methods

### Patients and samples

**Fig. 1:**
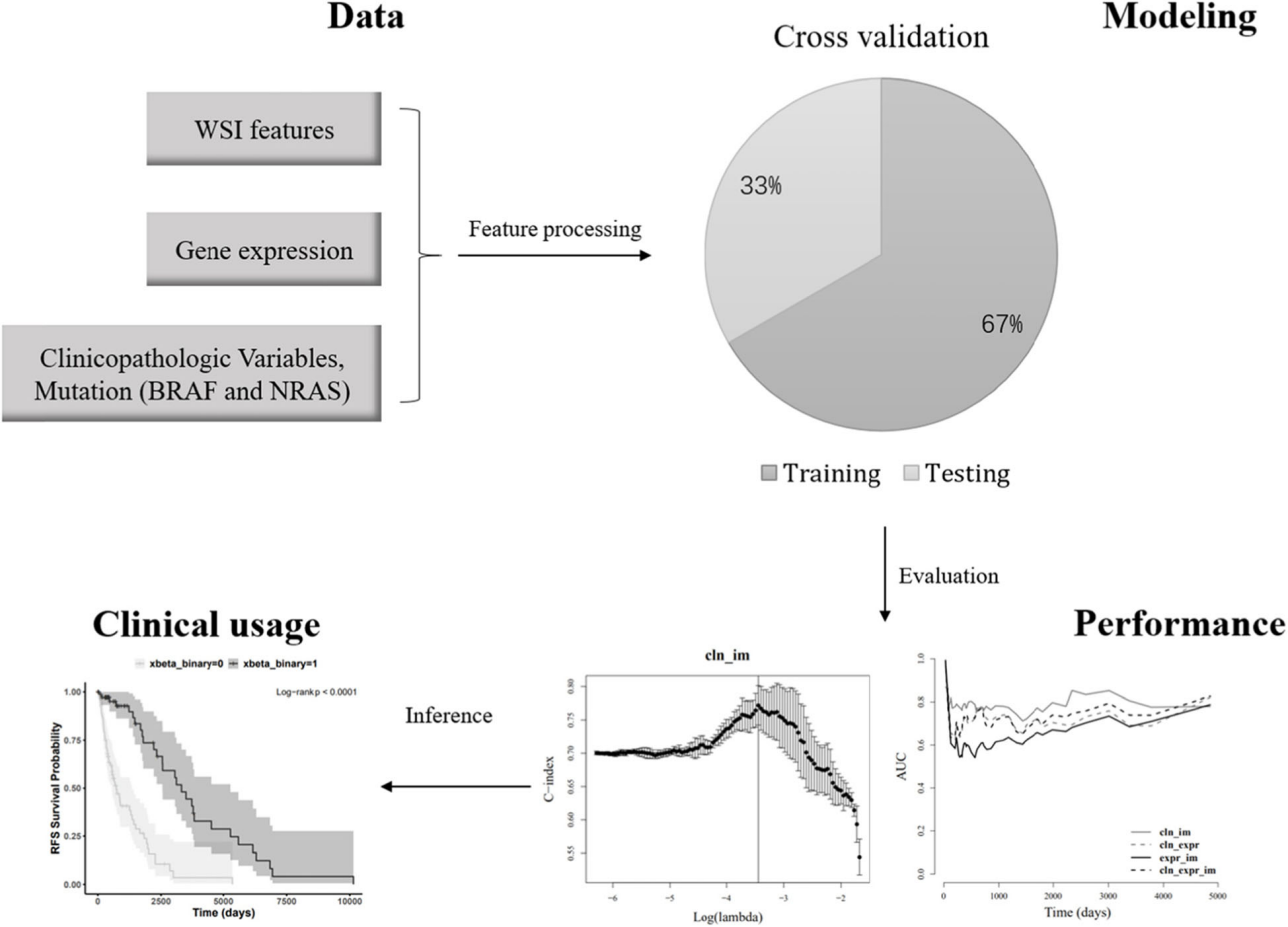
The workflow used in this study

This study was performed using 152 patients from the TCGA-SKCM [13] (The Cancer Genome Atlas Skin Cutaneous Melanoma) project. We enrolled patients that have complete information in AJCC stage, the dominant clinicopathologic variable used for RFS analysis (Additional file: Table S4). The enrolled patients should also have non-missing values in tumor location (metastatic or locoregional), WSI images, high-throughput gene expression data, and tumor recurrence-related follow-up. The study work flowchart is shown in Fig. 1. For each patient, the recurrence status was defined as 1 for those who had experienced recurrence and as 0 for censoring. The censoring time was set as the death time if one had the record of death or as last follow-up time if otherwise.

We collected 4 types of data for RFS analysis: clinicopathologic variables, high-throughput gene mutational profile, high-throughput gene expressional profile, and WSI features. For clinicopathologic variables, 4 covariates with complete records were included age at diagnosis, gender, primary location, and AJCC pathologic tumor stage. We selected the mutation status of NRAS and BRAF genes to represent the gene mutation data as previously described [7]. These clinicopathologic variables and gene mutations were used as the baseline variables for following model development. The distributions of these variables are summarized in Table 1. For gene expression, we used the FPKM (fragments per kilobase of exon model per million reads mapped) value to represent gene expression levels. A total of 5277 genes were selected for further analysis.

**Table 1:** Summary of distributions of baseline variables

Baseline variables	Summary
Age at diagnosis	mean = 60.06, std = 14.05
Gender	92 males
Primary location	60 metastatic, 92 locoregional
AJCC pathologic tumor stage	67 stage III and IV, 85 stage < III
BRAF	82 mutated
NRAS	32 mutated

### Whole slide image processing and feature extraction

All the melanoma tissue slides were stained by hematoxylin and eosin (H&E) and scanned by Aperio Digital Pathology Slide Scanner. Ten slides were magnified 20 times (× 20) and the other 142 slides were magnified 40 times (× 40). We extracted 3 types of texture features in 2 regions of interest (ROI): global and nucleus ROI, respectively. This process consisted of two steps: region segmentation and feature extraction. An illustration of these procedures is shown in Additional file: Figure S11. More details on the region segmentation can be found in the supplementary methods.

For feature extraction, we extracted 24 GLCM (gray level cooccurrence matrix) features, 16 GLRLM (gray level run length matrix) features, and 16 GLSZM (gray level size zone matrix) features [14] from each ROI. For nucleus ROIs from the same WSI, we calculated their mean, standard deviation, range, and disorder as the summary statistics [15]. This resulted in a total of 224 nucleus features and 56 global features. Image processing and feature extraction were performed by Python 3.7 and packages including “Pyradiomics” [14].

### Modeling and evaluation

We performed QR decomposition-based method [16] to reduce linear dependencies among the gene expressional profile before model fitting. In addition to the three types of features mentioned above, we also combined different types of features as new feature sets for model development. The number of features in each set is summarized in Additional file: Table S1.

We performed threefold cross validation for models developed based on each feature set. In each fold, a lasso Cox model was trained on the training set and was tested on the validation set. Due to the limited sample size, we used C-index computed from validation data to evaluate model performance. We also computed the time-dependent AUCs [17] from day 31 (5% percentile) to day 4631 (95% percentile) to evaluate the performance at each time point. The model development and comparison were performed by R version 4.1.

### Differential gene expression analysis

The differential gene expression analysis was performed using edgeR [18] package, and the gene ontology enrichment analysis was performed by GOseq [19] or clusterProfiler [20]. More details on the data processing and parameter setting can be found in the supplementary methods.

## Results

### Workflow and patient characteristic

The study workflow is shown in Fig. 1. We performed RFS modeling based on three types of variables: baseline variables (including clinicopathologic variables, and mutation status of NRAS and BRAF genes), gene expressional profile and WSI features. We developed 7 sets of features based on these three types of data (Additional file: Table S1). The model performance was evaluated by C-index and time-dependent AUC on threefold cross validation. We further presented the potential application of the best model under clinical setting. We also performed differential gene expression analysis between the higher and lower-risk subgroups stratified by our model for AJCC stage III patients.

We enrolled a total of 152 patients with complete information in recurrence-related survival time, baseline variables, gene expression data, and WSI features. A total of 82 patients had experienced recurrence, while 65 had last follow-up time and 5 died without recurrence. We performed Kaplan-Meier estimation for all patients and the RFS probability curve is shown in Additional file: Figure S2. The median survival time was 1757 days.

### Model comparisons

**Fig. 2:**
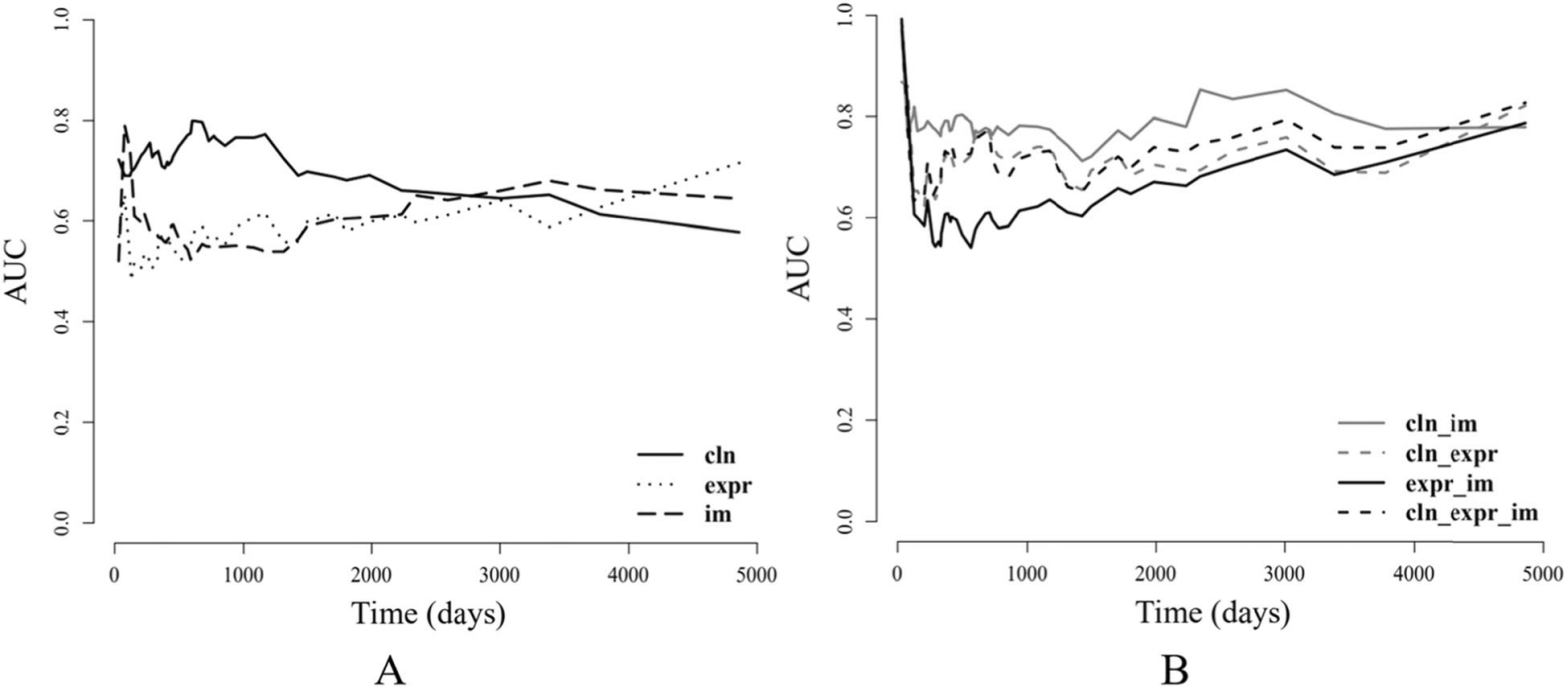
The time-dependent AUC of the models developed based on single-type or multi-type data. **a** Represents the time-dependent AUC of the models based on single-type data. **b** Represents the time-dependent AUC of the models based on multi-type data. Abbreviations: “cln”, baseline variables-based model; expr, models based on gene expression; “im”, models based on WSI features; “cln_im”, models based on baseline variables and WSI features; “cln_expr”, models based on baseline variables and gene expression; “expr_im”, models based on gene expression and WSI features; “cln_expr_im”, models based on baseline variables, gene expression, and WSI features

We compared the performance of models developed based on each single type of data to assess the prognostic power of single-type feature sets. The C-index of models based on baseline variables (mean/std = 0.654/0.014) was the highest (Additional file: Table S2, Figure S3). The models based on gene expression or WSI features had a slight difference (mean/std of C-index, expr = 0.639/0.039; im = 0.635/0.033). To evaluate the prediction accuracy at each time point, we computed the time-dependent AUC on the validation results. As shown in Fig. 2a, the models based on baseline variables showed obvious superiority until about day 2500. In contrast, the model based on WSI features had increasing prediction accuracy since about day 1500. This motivated the combinatorial modeling, as combing baseline and WSI feature in survival prediction might utilize the prediction advantage of single-type data-based model within specific time intervals.

We then developed models based on combinations of different types of data, and compared their prediction performance. For WSI image analysis, we extracted texture features from global regions or segmented nucleus regions (Additional file: Figure S1). As summarized in Additional file: Tables S2, the best C-index (mean/std = 0.772/0.029) was achieved by the model combining baseline variables and WSI features (Additional file: Figure S3). As shown in Additional file: Figure 2B, such model also had the best performance at almost every time point as measured by time-dependent AUC (mean/std of time-dependent AUC = 0.785/0.038), indicating a clear benefit of data integration. We therefore used this feature set and the optimal penalty (Additional file: Figure S3) to develop a lasso Cox model on all patients. This model was used as the final prognostic model, and the coefficients of selected features were showed in Additional file: Table S3.

### The image-based prognostic model

The proposed model included 20 WSI features and 5 baseline variables. For the WSI features, 14 of them were extracted from nucleus ROIs and 6 were from global ROIs. The computational formula of each feature was shown in supplementary methods. A positive value of coefficient represented that the hazard of recurrence would increase with the feature values. As shown in Additional file: Table S3, the 3 largest absolute values of the coefficients were from GLRLM (*RunEntropy_std* and *ShortRunEmphasis_range*) and GLCM (*Idn_range*) in nucleus ROIs. Both the *RunEntropy* and *ShortRunEmphasis* were the measurement of the distribution of run lengths. The *Idn* (inverse difference normalized) quantified the local homogeneity within nucleus ROI, which could be low value if there was necrosis or dissolution of nucleus. Since the standard deviation or range of these features was significant predictor in the model, we inferred that the variance of nuclei shape, surrounding textures, and homogeneity contributed to an effective prognostic analysis. For the features extracted from global ROIs, the *glcm_Idmn* and *glszm_LargeAreaHighGrayLevelEmphasis* had the largest absolute coefficients. The *Idmn* (inverse difference moment normalized) also measured the local homogeneity, while the *LargeAreaHighGrayLevelEmphasis* computed the emphasis of regions with large area and high gray level. Both of the features were indicators of the tumor region size.

### Risk stratification

To illustrate potential applications of the prognostic model, we computed a risk score by summing over the product of model features and their coefficients. To compare the risk score with traditional prognostic variables, we also computed risk scores for models based on AJCC pathologic tumor stage or baseline variables. We used each risk score to fit a univariate Cox PH model on 5 subgroups of patients (all, AJCC stage < III, AJCC stage ≥ III, metastatic or locoregional) and applied likelihood ratio test to assess the significance of the score. As summarized in Additional file: Table S4, both the likelihood ratio test statistic and its *p* value showed that adding WSI features to baseline variables greatly improved the prediction accuracy.

**Fig. 3:**
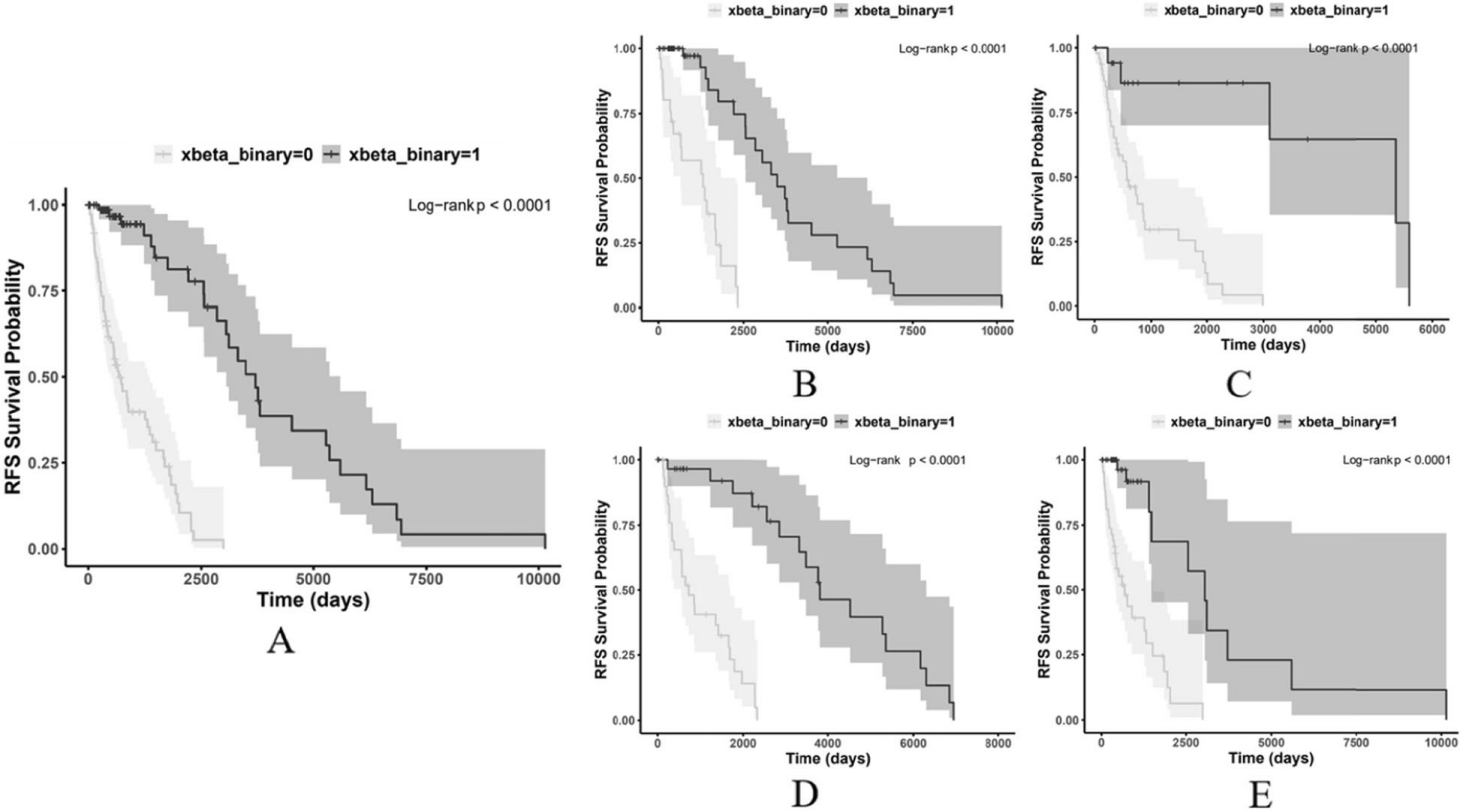
The recurrence-free survival probability of subgroups stratified by the risk score. **a** Represents all the patients. **b** For those in AJCC stage < III. **c** For those in AJCC stage ≥ III. **d** For those with metastatic tumors. **e** For those with locoregional tumors

To illustrate the independent prognostic value of image-based risk score, we set the median of risk score as the threshold and stratified all the patients into a higher or lower-risk group. We then performed Kaplan-Meier estimation for each group. As shown in Fig. 3a, the survival distributions of the two risk groups characterized among all patients were significantly different (log-rank *p* value < 0.0001). The median survival time of the two risk groups were 678 days and 3716 days, respectively. We also estimated the survival probability within 4 specific pathologically-defined subgroups of patients (Fig. 3). The survival distributions were all significantly different for each subgroup. The median survival time of each subgroup was shown in Additional file: Table S5. Of note, the lower-risk group in patients with severe stage (AJCC stage ≥ III) had a median RFS time of 5354 days, which is 1.54 times longer than that of with mild or moderate stage (AJCC stage < III), who had a median RFS time of 3488 days. As shown in Additional file: Figure S4, this risk score could significantly stratify the higher and lower-risk group for overall survival as well.

### Differential risk-related enrichment of gene sets

To explore the molecular mechanisms underlying the superiority of image-based prognostic model, we performed differential gene expression analysis between the higher (43 patients) and the lower-risk (15 patients) group characterized by our model for patients within AJCC stage III. To reduce the biological variation within each risk group, we calculated the Spearman correlation coefficients between any pairs of samples and filtered out those with a correlation coefficient smaller than 0.85 with more than 20 other samples among the higher-risk group, or with more than 7 other samples among the lower-risk group. This resulted in 9 lower-risk patients and 21 higher-risk patients for further analyses.

A total of 188 downregulated and 28 upregulated genes were identified as significantly differentially expressed. We found 226 enriched BP (biological process), 18 enriched CC (cellular component), and 12 MF (molecular function) GO terms. As shown in Additional file: Figure S5, the most majority of top 20 enriched BP terms was involved in immune response, immune cell activation and proliferation. In particular, the T cell activation and proliferation-related pathways were significantly enriched. These enriched BP terms were also identified by the clusterProfiler package as shown in Additional file: Figure S8, which suggested that the variation in the regulation of T cell activation was the potential key driver for differential risk. For CC terms, the T cell receptor-related GO terms were significantly enriched as shown in Additional file: Figure S6 and S9. For MF terms, MHC protein binding and cytokine activity-related GO were associated with the risk stratification (as shown in Additional file: Figure S7 and S10). These enriched GO terms suggested that the selected image features accounted for the variations of T cell activities and hence provided a more accurate assessment of disease progression.

## Discussion

In this study, we performed recurrence-free survival analysis, and developed lasso Cox prediction models based on different types of data (baseline variables, gene expression, and whole slide image features). Accurate RFS measurements have become increasingly important as the treatment of cutaneous melanoma has significantly improved over years. A reliable RFS prediction could thus assist in more precision treatment selection for melanoma patients. Our evaluation criteria included C-index and time-dependent AUC. We identified that combining baseline variables and WSI features achieved the best prediction performance (cross validation C-index, 0.772/0.029; time-dependent AUC, 0.785/0.038). We showed that models trained on single-type data could have varied prediction accuracy within specific time intervals. We were then motivated to combine different types of data to develop a model that achieved uniformly best prediction accuracy at most majority of time after initial diagnosis. We also showed that this combinatorial model provided significant risk stratifications within specific subgroups defined by metastatic status or AJCC pathological tumor stages. We found from gene expressional profiles that T cell activities were significantly associated with the differential risk determined by the image-based prognostic model.

T cell activation is closely related to the adoptive or targeted immunotherapies. Such immunotherapies either provide co-stimulatory signals to trigger T cell proliferation, or block inhibitory molecules to unleash the anti-tumor T cell activities [21]. Our findings indicate that, even within specific risk groups (e.g., AJCC stage III) as defined by routine clinicopathological variables, it is still possible to further identify subgroups characterized with the variation in T cell activation using WSI features. Therefore, whether such subgroups have differential responses to immunotherapy, such as PD-1 or CTLA4-based immune checkpoint therapies, warrants further investigation.

Our study provided a cost-effective way to evaluate the prognosis by only taking baseline variables and automatically generated WSI features as the input. We showed that many top enriched GO terms were related to immune response, T cell activation and proliferation. Genes related to T cell development and function were also identified by Jiang et al. [8] as significantly associated with overall survival by analyses performed based on clinicopathologic variables, methylation, CNA (copy number alteration), gene expression, and mutational data. Pastorfide et al. [21] and others [22, 23] also proved that lymphocytic infiltrates were associated with metastasis and survival outcome by artificial analyses of histologic images. These findings were consistent with our study, but we used computer-aided image processing instead to automatically quantify such information from routinely made WSI.

Our study has limitations. First, due to a large number of missing values, we only enrolled 152 patients with complete information in our study. The best model was determined by threefold cross validation without being tested on an independent dataset. Second, the quality of pathology images varied. The variation in staining intensity and marker-pen pollution were the two most frequent problems. The former problem could affect nucleus segmentation, and we mitigated this by segmenting ROIs centering around the localized nucleus region. We also manually checked each ROI to remove polluted ones. Third, we did not evaluate the effects of interaction between different types of data when performing data integration. Fourth, we did not include some other routinely used prognostic variables, such as Breslow tumor thickness, ulceration, mitotic rate, and Clark levels, due to a large proportion of missing values (Additional file: Table S8). These variables might improve the prognostic prediction performance of our models.

For the future extension of our study, we will perform external validation of the prediction model using multi-center retrospective cohort data. Another key point that needs to be addressed in future studies is the assessment of effect of clinicopathological variables and treatment on RFS prediction using more complete data. Notwithstanding these limitations, we believe our method has potential for clinical translation to reduce the level of heterogeneity in RFS for cutaneous melanoma patients. This could influence the treatment selection for patients, as well as the patient enrollment for related clinical trials. Moreover, considering the capacity of WSI texture features in capturing immune cell activity in this study, we believe that WSI texture features could also have prognostic values in other types of cancer, which warrants exploration in future studies.

## Conclusions

In summary, we developed an image-based combinatorial model and demonstrated its prediction ability for recurrence-free survival. The model includes 20 automatically generated WSI features, 3 clinicopathologic variables, and mutation status of 2 genes. We hence provided a cost-effective prognostic model as a substitute for gene expression profiling-based prognosis methods.

## Supplementary information

**Additional file 1: Table S1.** The composition and number of features in each feature set. **Table S2.** Summary of C-index and time-dependent AUC. **Table S3.** The name and coefficient of features selected in the final image-based model. **Table S4.** The likelihood ratio (LR) and its p value of models. **Table S5.** The median survival time of higher and lower-risk subgroups in each pathologically-defined groups of patients. **Table S6.** Summary of treatment information and their RFS associations of the study cohort. **Table S7.** Summary of therapeutics type among the 50 patients with pharmaceutical treatment information available. **Table S8.** Summary of some omitted clinicopathologic variables routinely used for prognostic analysis in the study cohort. **Figure S1.** Three examples of nucleus segmentation results. **Figure S2.** The RFS probability curve of the 152 patients enrolled in this study. **Figure S3.** Analysis of variation of cross-validation C-index along with the penalty (log-transformed *λ*). **Figure S4.** The overall survival probability of subgroups stratified by the risk score. **Figure S5.** The dot plot of the top 20 GO in BP identified by GOseq package. **Figure S6.** The dot plot of the top 20 gene ontologies in CC identified by GOseq package. **Figure S7.** The dot plot of the top 20 gene ontologies in MF identified by GOseq package. **Figure S8.** The directed acyclic graph of the enriched GO terms in biological process category identified by clusterProfiler package. **Figure S9.** The directed acyclic graph of the enriched GO terms in cellular component category identified by clusterProfiler package. **Figure S10.** The directed acyclic graph of the enriched GO terms in molecular function category identified by clusterProfiler package. **Figure S11.** An illustration of WSI processing and feature extraction.

## Data Availability

The datasets analyzed during the current study are available in the TCGA-SKCM repository, https://portal.gdc.cancer.gov/projects/TCGA-SKCM.

## Abbreviations

RFS: Recurrence-free survival
WSI: Whole slide image
AJCC: The American Joint Committee on Cancer
TCGA-SKCM: The Cancer Genome Atlas Skin Cutaneous Melanoma
FPKM: Fragments per kilobase of exon model per million reads mapped
H&E: Hematoxylin and eosin
ROI: Region of interest
GLCM: Gray level cooccurrence matrix
GLRLM: Gray level run length matrix
GLSZM: Gray level size zone matrix
TMM: The trimmed mean of *M* values
AUC: Area under the curve
FDR: False discovery rate
GO: Gene ontology
BP: Biological process
CC: Cellular component
MF: Molecular function
CNA: Copy number alteration
Idn: Inverse difference normalized
Idmn: Inverse difference moment normalized
cln: Clinicopathologic variables, mutation status of BRAF, and NRAS genes
expr: The selected gene expression data
im: Whole slide image features
cln_expr: The combination of cln and expr
cln_im: The combination of cln and im
expr_im: The combination of expr and im
cln_expr_im: The combination of cln, expr, and im

## References

[1] Bray F, Ferlay J, Soerjomataram I, Siegel RL, Torre LA, Jemal A (2018). Global cancer statistics 2018: GLOBOCAN estimates of incidence and mortality worldwide for 36 cancers in 185 countries. CA Cancer J Clin.

[2] Ali Z, Yousaf N, Larkin J (2013). Melanoma epidemiology, biology and prognosis. EJC Suppl.

[3] Chang AE, Karnell LH, Menck HR (1998). The National Cancer Data Base report on cutaneous and noncutaneous melanoma: a summary of 84,836 cases from the past decade. The American College of Surgeons Commission on Cancer and the American Cancer Society. Cancer..

[4] Dickson PV, Gershenwald JE (2011). Staging and prognosis of cutaneous melanoma. Surg Oncol Clin N Am.

[5] Hyams DM, Cook RW, Buzaid AC (2019). Identification of risk in cutaneous melanoma patients: prognostic and predictive markers. J Surg Oncol.

[6] Zhao Y, Schaafsma E, Gorlov IP, Hernando E, Thomas NE, Shen R (2019). A leukocyte infiltration score defined by a gene signature predicts melanoma patient prognosis. Mol Cancer Res.

[7] Jayawardana K, Schramm SJ, Haydu L, Thompson JF, Scolyer RA, Mann GJ (2015). Determination of prognosis in metastatic melanoma through integration of clinico-pathologic, mutation, mRNA, microRNA, and protein information. Int J Cancer.

[8] Jiang Y, Shi X, Zhao Q, Krauthammer M, Rothberg BE, Ma S (2016). Integrated analysis of multidimensional omics data on cutaneous melanoma prognosis. Genomics..

[9] Lu C, Mandal M (2015). Automated analysis and diagnosis of skin melanoma on whole slide histopathological images. Pattern Recogn.

[10] Failmezger H, Muralidhar S, Rullan A, de Andrea CE, Sahai E, Yuan Y (2020). Topological tumor raphs: a graph-based spatial model to infer stromal recruitment for immunosuppression in melanoma histology. Cancer Res.

[11] Robert C, Thomas L, Bondarenko I, O’Day S, Weber J, Garbe C (2011). Ipilimumab plus dacarbazine for previously untreated metastatic melanoma. N Engl J Med.

[12] Robert C, Schachter J, Long GV, Arance A, Grob JJ, Mortier L (2015). Pembrolizumab versus ipilimumab in advanced melanoma. N Engl J Med.

[13] Cancer Genome Atlas N (2015). Genomic classification of cutaneous melanoma. Cell..

[14] van Griethuysen JJM, Fedorov A, Parmar C, Hosny A, Aucoin N, Narayan V (2017). Computational radiomics system to decode the radiographic phenotype. Cancer Res.

[15] Doyle S, Feldman MD, Shih N, Tomaszewski J, Madabhushi A (2012). Cascaded discrimination of normal, abnormal, and confounder classes in histopathology: Gleason grading of prostate cancer. BMC Bioinformatics.

[16] Jones S YZ, Xie Z, et al. A proposed data analytics workflow and example using the R caret package.

[17] Heagerty PJ, Lumley T, Pepe MS (2000). Time-dependent ROC curves for censored survival data and a diagnostic marker. Biometrics..

[18] Robinson MD, McCarthy DJ, Smyth GK (2010). edgeR: a bioconductor package for differential expression analysis of digital gene expression data. Bioinformatics..

[19] Young MD, Wakefield MJ, Smyth GK, Oshlack A (2010). Gene ontology analysis for RNA-seq: accounting for selection bias. Genome Biol.

[20] Yu G, Wang LG, Han Y, He QY (2012). clusterProfiler: an R package for comparing biological themes among gene clusters. OMICS..

[21] Pastorfide GC, Kibbi AG, de Roa AL, Barnhill RL, Sober AJ, Mihm MC (1992). Image analysis of stage 1 melanoma (1.00-2.50 mm): lymphocytic infiltrates related to metastasis and survival. J Cutan Pathol.

[22] Kornstein MJ, Brooks JS, Elder DE (1983). Immunoperoxidase localization of lymphocyte subsets in the host response to melanoma and nevi. Cancer Res.

[23] Ralfkiaer E, Hou-Jensen K, Gatter KC, Drzewiecki KT, Mason DY (1987). Immunohistological analysis of the lymphoid infiltrate in cutaneous malignant melanomas. Virchows Arch A Pathol Anat Histopathol.

