# Supplementary material for: Combining texture features of whole slide images improves prognostic prediction of recurrence-free survival for cutaneous melanoma patients
Source: World J Surg Oncol. 2020 Jun 16;18:130. doi: 10.1186/s12957-020-01909-5 (PMC7298832; doi:10.1186/s12957-020-01909-5)
Supplement: Supplementary file 1 — Additional file 1: Table S1. The composition and number of features in each feature set. Table S2. Summary of C-index and time-dependent AUC. Table S3. The name and coefficient of features selected in the final image-based model. Table S4. The likelihood ratio (LR) and its p value of models. Table S5. The median survival time of higher and lower-risk subgroups in each pathologically-defined groups of patients. Table S6. Summary of treatment information and their RFS associations of the study cohort. Table S7. Summary of therapeutics type among the 50 patients with pharmaceutical treatment information available. Table S8. Summary of some omitted clinicopathologic variables routinely used for prognostic analysis in the study cohort. Figure S1. Three examples of nucleus segmentation results. Figure S2. The RFS probability curve of the 152 patients enrolled in this study. Figure S3. Analysis of variation of cross-validation C-index along with the penalty (log-transformed λ). Figure S4. The overall survival probability of subgroups stratified by the risk score. Figure S5. The dot plot of the top 20 GO in BP identified by GOseq package. Figure S6. The dot plot of the top 20 gene ontologies in CC identified by GOseq package. Figure S7. The dot plot of the top 20 gene ontologies in MF identified by GOseq package. Figure S8. The directed acyclic graph of the enriched GO terms in biological process category identified by clusterProfiler package. Figure S9. The directed acyclic graph of the enriched GO terms in cellular component category identified by clusterProfiler package. Figure S10. The directed acyclic graph of the enriched GO terms in molecular function category identified by clusterProfiler package. Figure S11. An illustration of WSI processing and feature extraction. [file 12957_2020_1909_MOESM1_ESM.docx]

**Supplementary materials**

**Combining texture features of whole slide images improves prognostic prediction of recurrence-free survival for cutaneous melanoma patients**

**Yanbin Peng^1^, Yunfeng Chu^1^, Zhong Chen^1^, Wen Zhou^1^, Shengxiang Wan^1^, Yingfeng Xiao^1^, Youlong Zhang^2^, Jialu Li^2^**

1 Department of Microsurgery, Peking University Shenzhen Hospital

2 Department of Biostatistics, HuaJia Biomedical Intelligence

*Contributions:* (I) Conception and design: Yanbin Peng, Jialu Li; (II) Administrative support: Jialu Li; (III) Collection and assembly of data: Yanbin Peng, Youlong Zhang; (IV) Data analysis and interpretation: Yanbin Peng, Youlong Zhang, Jialu Li; (V) Manuscript writing: All authors; (VI) Final approval of manuscript: All authors.

*Correspondence to:* Jialu Li, PhD, Department of Biostatistics, HuaJia Biomedical Intelligence, Shenzhen Overseas Chinese High-Tech Venture Park, Nanshan district, Shenzhen, China, 518057. Email: [Jialu.li@huajiabio.com](mailto:Jialu.li@huajiabio.com)

Contents

[Supplementary Tables 1](#_Toc41503235)

[Supplementary Figures 8](#_Toc41503236)

[Supplementary Methods 19](#_Toc41503237)

# Supplementary Tables

Table S1: The composition and number of features in each feature set.

| Feature sets | Description | Number |
| --- | --- | --- |
| cln | clinicopathologic variables, BRAF and NRAS | 6 |
| expr | selected gene expression data | 152 |
| im | whole slide image features | 280 |
| cln_expr | cln and expr | 158 |
| cln_im | cln and im | 286 |
| expr_im | expr and im | 432 |
| cln_expr_im | cln, expr and im | 438 |

Table S2: Summary of C-index and time-dependent AUC.

| Feature sets | C-index | | time-dependent AUC | |
| --- | --- | --- | --- | --- |
|  | mean | std | mean | std |
| cln | 0.654 | 0.014 | 0.720 | 0.048 |
| expr | 0.639 | 0.039 | 0.570 | 0.043 |
| im | 0.635 | 0.033 | 0.590 | 0.058 |
| cln_expr | 0.651 | 0.018 | 0.715 | 0.051 |
| cln_im | **0.772** | 0.029 | **0.785** | 0.038 |
| expr_im | 0.590 | 0.050 | 0.628 | 0.081 |
| cln_expr_im | 0.618 | 0.016 | 0.721 | 0.060 |

Table S3: The name and coefficient of features selected in the final image-based model.

| Names | Coefficients |
| --- | --- |
| age_at_diagnosis | 0.205 |
| gender | 0.195 |
| ajcc_pathologic_tumor_stage | 0.958 |
| BRAF | 0.144 |
| NRAS | 0.032 |
| nu_glcm_ClusterShade_mean | -0.158 |
| nu_glcm_ClusterShade_range | 0.029 |
| nu_glcm_DifferenceVariance_range | -0.097 |
| nu_glcm_Idn_range | 0.580 |
| nu_glcm_InverseVariance_std | -0.176 |
| nu_glrlm_GrayLevelNonUniformity_range | -0.010 |
| nu_glrlm_RunEntropy_range | 0.352 |
| nu_glrlm_RunEntropy_std | 0.627 |
| nu_glrlm_ShortRunEmphasis_range | -0.621 |
| nu_glrlm_ShortRunLowGrayLevelEmphasis_mean | -0.572 |
| nu_glrlm_ShortRunLowGrayLevelEmphasis_range | 0.167 |
| nu_glrlm_ShortRunLowGrayLevelEmphasis_std | -0.192 |
| nu_glszm_LargeAreaHighGrayLevelEmphasis_disorder | -0.110 |
| nu_glszm_SizeZoneNonUniformityNormalized_disorder | 0.042 |
| global_glcm_Idmn | 0.308 |
| global_glcm_InverseVariance | 0.034 |
| global_glcm_MaximumProbability | -0.147 |
| global_glrlm_GrayLevelNonUniformity | 0.194 |
| global_glszm_GrayLevelNonUniformityNormalized | -0.034 |
| global_glszm_LargeAreaHighGrayLevelEmphasis | -0.206 |

Table S4: The likelihood ratio (LR) and its p value of models.

| Groups | AJCC stage | | cln | | cln_im | |
| --- | --- | --- | --- | --- | --- | --- |
|  | LR | p value | LR | p value | LR | p value |
| All | 11.016 | <0.001 | 18.203 | <0.001 | 118.641 | 1.25E-27 |
| AJCC stage<III | / | / | 4.752 | 0.029 | 46.558 | 8.89E-12 |
| AJCC stage≥III | / | / | 2.130 | 0.144 | 52.776 | 3.74E-13 |
| Metastatic | 6.394 | 0.011 | 9.911 | 0.002 | 57.115 | 4.11E-14 |
| Locoregional | 3.651 | 0.056 | 6.395 | 0.011 | 54.167 | 1.84E-13 |

“AJCC stage”, “cln” and "cln_im” represent models based on AJCC tumor pathologic stage, baseline variables and the combination of baseline variables and WSI features, respectively. Abbreviations: “All”, all the patients; “AJCC stage<III”, patients within AJCC tumor pathologic stage<III group; “AJCC stage≥III”, patients within AJCC tumor pathologic stage≥III group; “Metastatic”, the group of patients with metastatic tumors; “Locoregional”, the group of patients with locoregional tumors.

Table S5: The median survival time of higher and lower-risk subgroups in each pathologically-defined groups of patients.

| groups | higher-risk | | lower-risk |
| --- | --- | --- | --- |
| All | 678 | 3716 | |
| AJCC stage<III | 1308 | 3488 | |
| AJCC stage≥III | 570 | 5354 | |
| Metastatic | 728 | 3812 | |
| Locoregional | 678 | 3050 | |

Table S6: Summary of treatment information and their RFS associations of the study cohort.

| Variables | Distribution | p-value (univariate model) | p-value (multivariate model)† |
| --- | --- | --- | --- |
| neo-adjuvant | 147 no, 5 yes | 0.623 | / |
| radiation | 117 no, 31 yes, 4 na § | 0.033 | 0.124 |
| pharmaceutical | 99 no, 50 yes, 3 na | 0.12 | / |

† The multivariate model includes all six variables (age, gender, AJCC stage, primary location, BRAF and NRAS mutation status) used in the “cln” model. Only variables with a significant p-value under univariate model proceeded to multivariate analysis.

§ For the multivariate modeling, the missing value was imputed with binomial sampling with a probability of 31/(117+31)= 0.21.

Table S7: Summary of therapeutics type among the 50 patients with pharmaceutical treatment information available.

| Variables | Distribution | p-value (RFS univariate model) | 2x2 table with the risk score † | | Fisher exact test p-value of the table |
| --- | --- | --- | --- | --- | --- |
|  |  |  | High (n=33) | Low (n=17) |  |
| Only chemotherapy | 37 no | 0.0113 | 23 | 14 | 0.499 |
|  | 13 yes |  | 10 | 3 |  |
| Only immunotherapy § | 31 no | 0.0114 | 23 | 8 | 0.137 |
|  | 19 yes |  | 10 | 9 |  |
| Both chemotherapy and immunotherapy | 41 no | 0.148 | 26 | 15 | 0.699 |
|  | 9 yes |  | 7 | 2 |  |
| No chemotherapy or immunotherapy | 41 no | 0.815 | 27 | 14 | 1 |
|  | 9 yes |  | 6 | 3 |  |

† The median of the risk scores computed from the 152 patients data was used as the threshold here to stratify high-risk from low-risk group.

§ This includes the usage of monoclonal antibody (Ipilimumab or Pembrolizumab), interferon or Interleukin.

Table S8: Summary of some omitted clinicopathologic variables routinely used for prognostic analysis in the study cohort.

| Variables | Distribution (total sample size=152) | p-value (RFS univariate model) |
| --- | --- | --- |
| Breslow thickness | n=122 † | 0.190 |
| mean/std. | 8.16/12.12 |  |
| primary melanoma tumor ulceration | n=111 | 0.030 |
| yes | 70 |  |
| primary melanoma mitotic rate | n=44 | 0.236 |
| mean/std. | 5.11/4.13 |  |
| Clark level at diagnosis | n=99 | 0.065 |
| level II | 6 | 0.934 |
| level III | 22 | 0.041 |
| level IV | 55 | 0.190 |
| level V | 16 | 0.173 |

† This indicates the number of patients with complete information of such clinicopathologic variable.

# Supplementary Figures


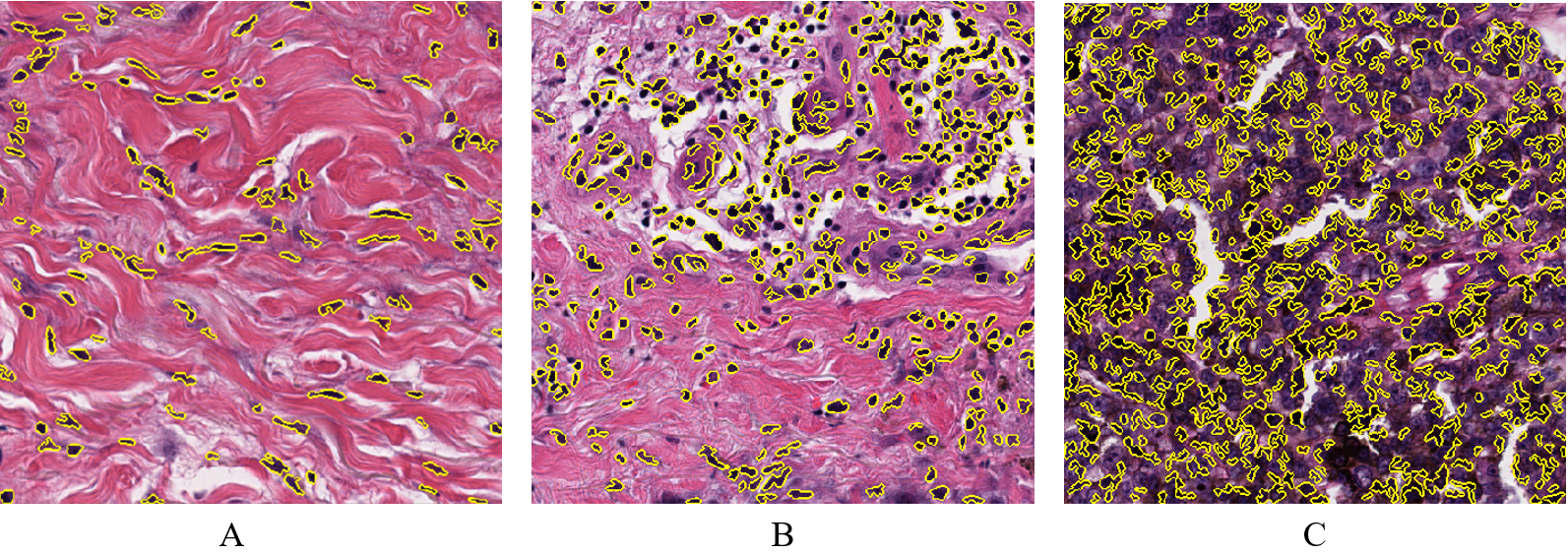


Figure S1: Three examples of nucleus segmentation results. Figure A shows an image block with a small number of nuclei; B is a block with a higher number of nuclei; C is a block almost all filled with nuclei.


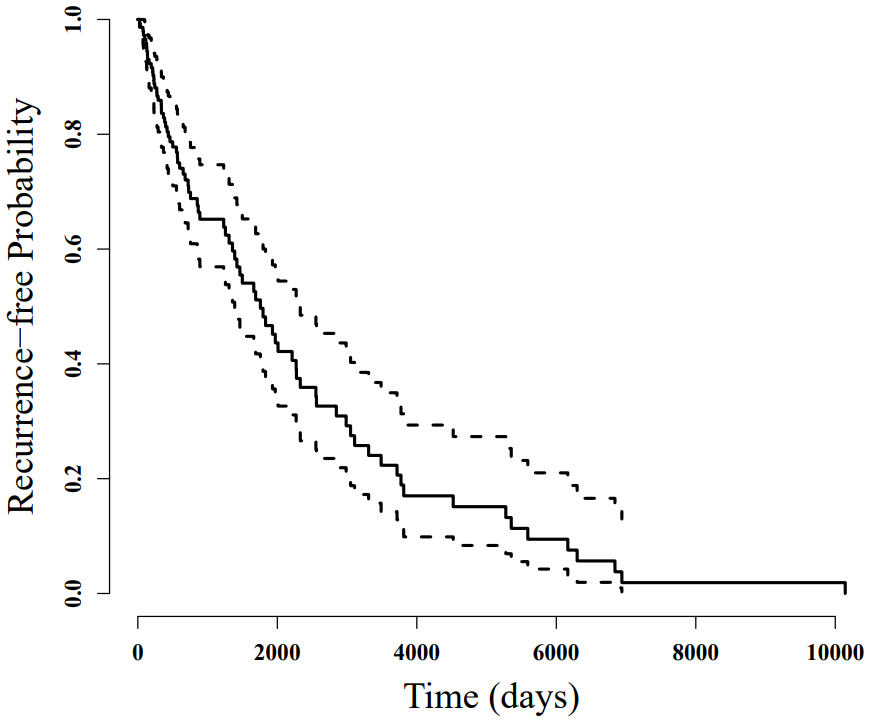


Figure S2: The RFS probability curve of the 152 patients enrolled in this study.


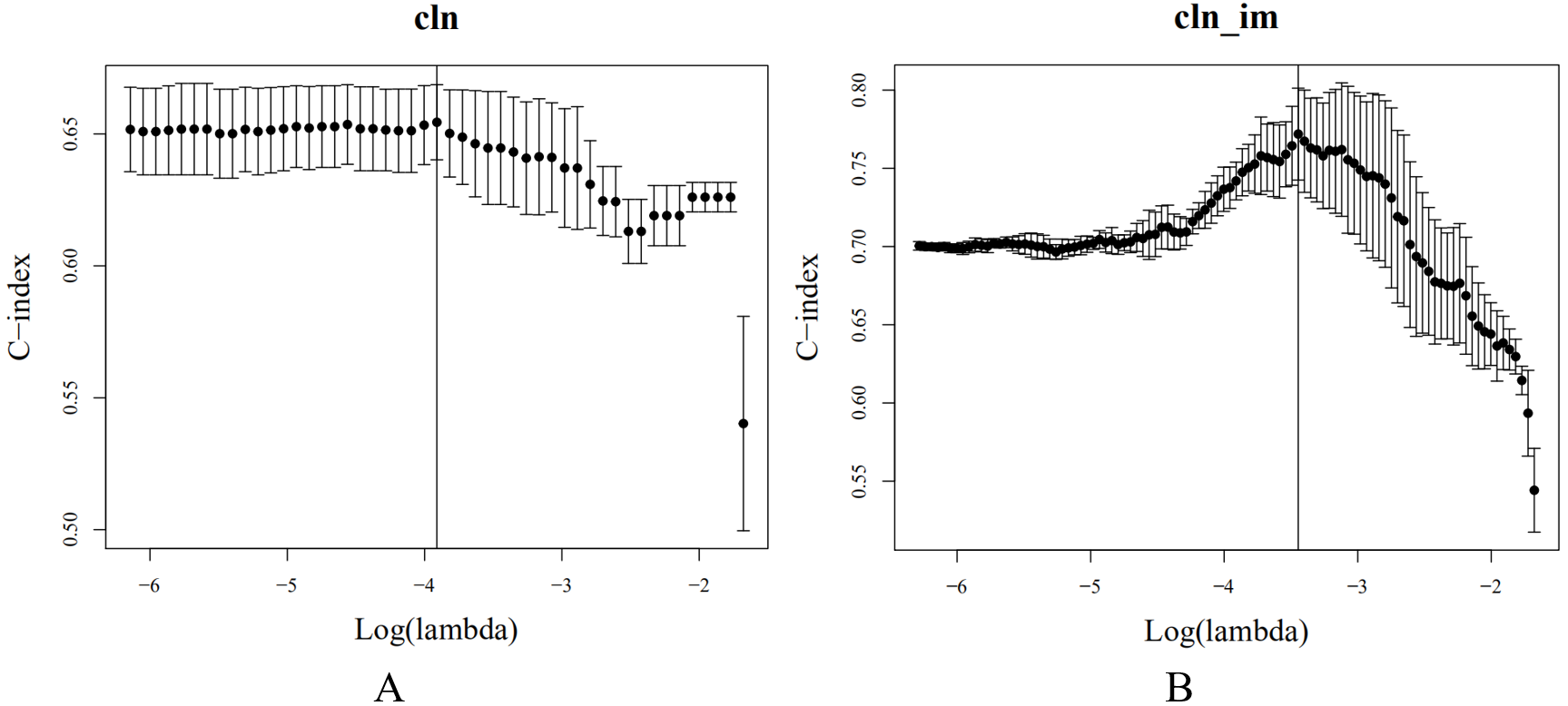


Figure S3: Analysis of variation of cross-validation C-index along with the penalty (log-transformed $\lambda$). Figure A was for models developed based on baseline variables, while figure B for that of based on both baseline variables and WSI features.


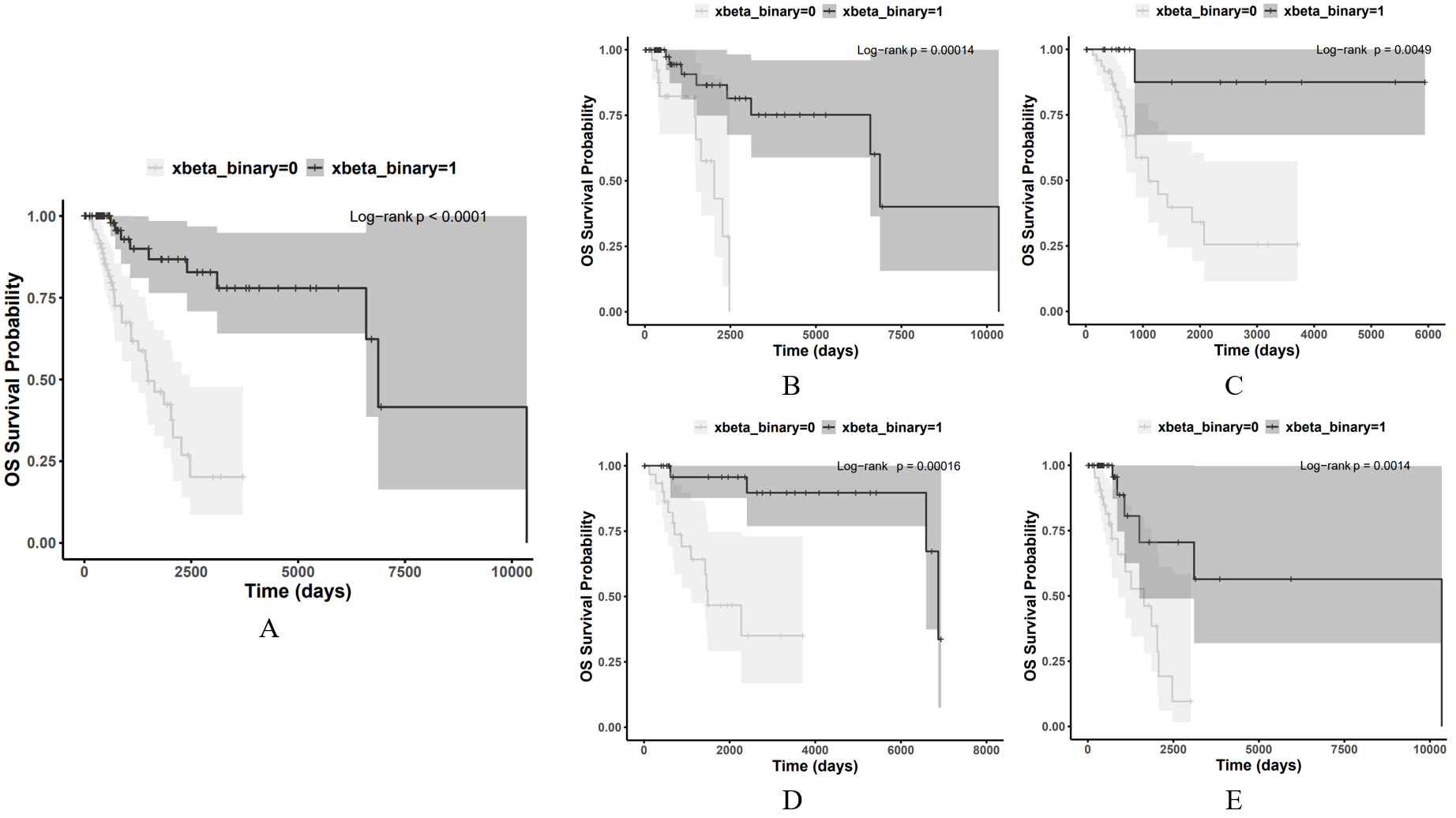


Figure S4: The overall survival probability of subgroups stratified by the risk score. A represents all the patients; B represents the patients in AJCC stage<III; C represents the patients in AJCC stage≥III; D represents the patients with metastatic tumors; E represents the patients with locoregional tumors.


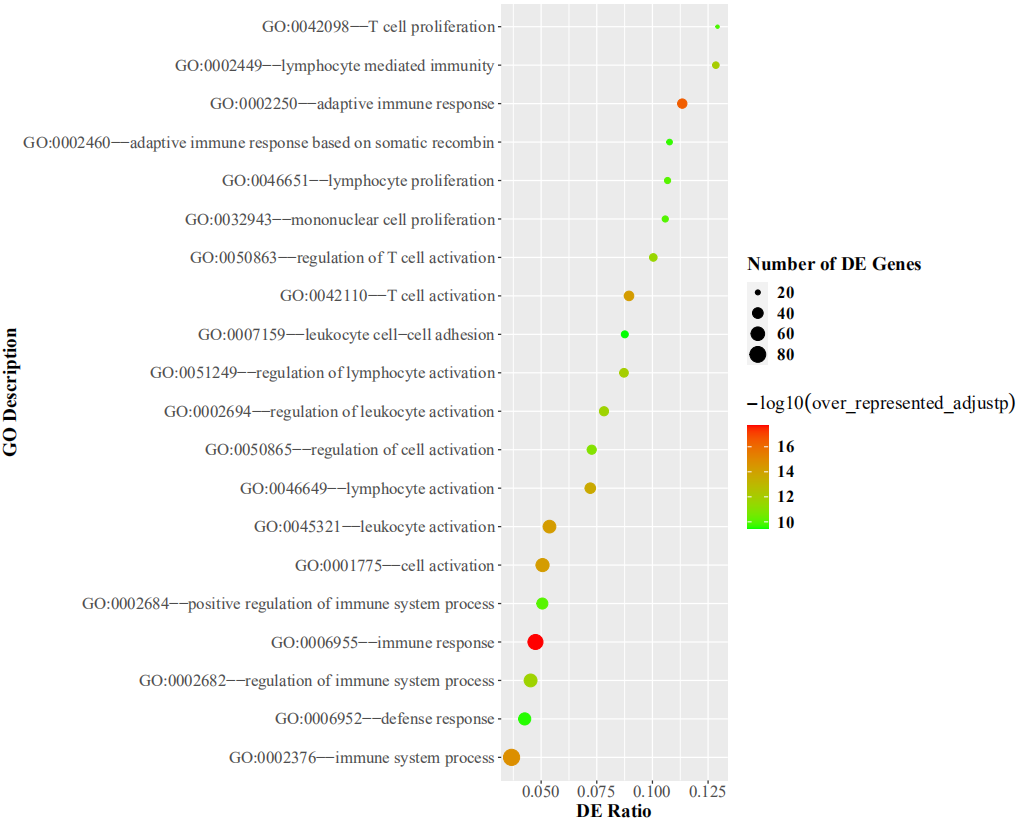


Figure S5: The dot plot of the top 20 GO in BP identified by GOseq package. The DE Ratio is the ratio of differentially expressed genes among all the genes in a specific GO category. The GO Description displays the ID and brief information of each GO. The color of dot shows the adjusted p value of the GO term. The size of dot represents the number of differentially expressed genes.


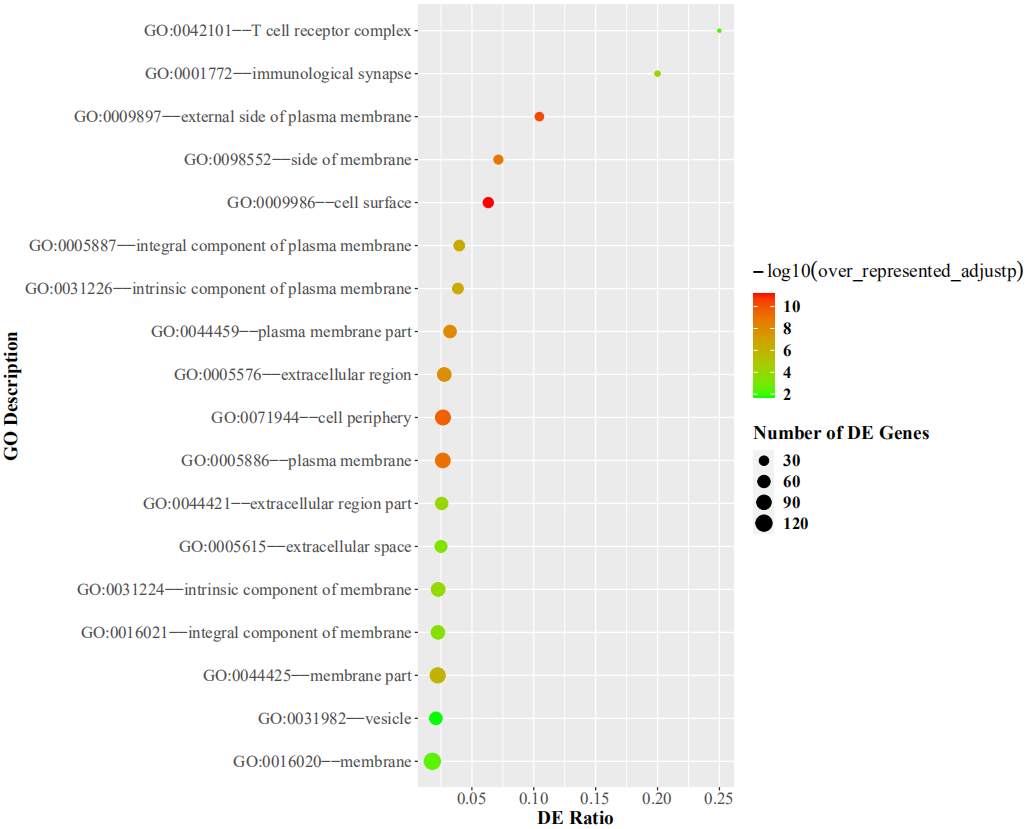


Figure S6: The dot plot of the top 20 gene ontologies in CC identified by GOseq package. The DE Ratio is the ratio of differentially expressed genes among all the genes in a specific GO category. The GO Description displays the ID and brief information of each GO. The color of dot shows the adjusted p value of the GO term. The size of dot represents the number of differentially expressed genes.


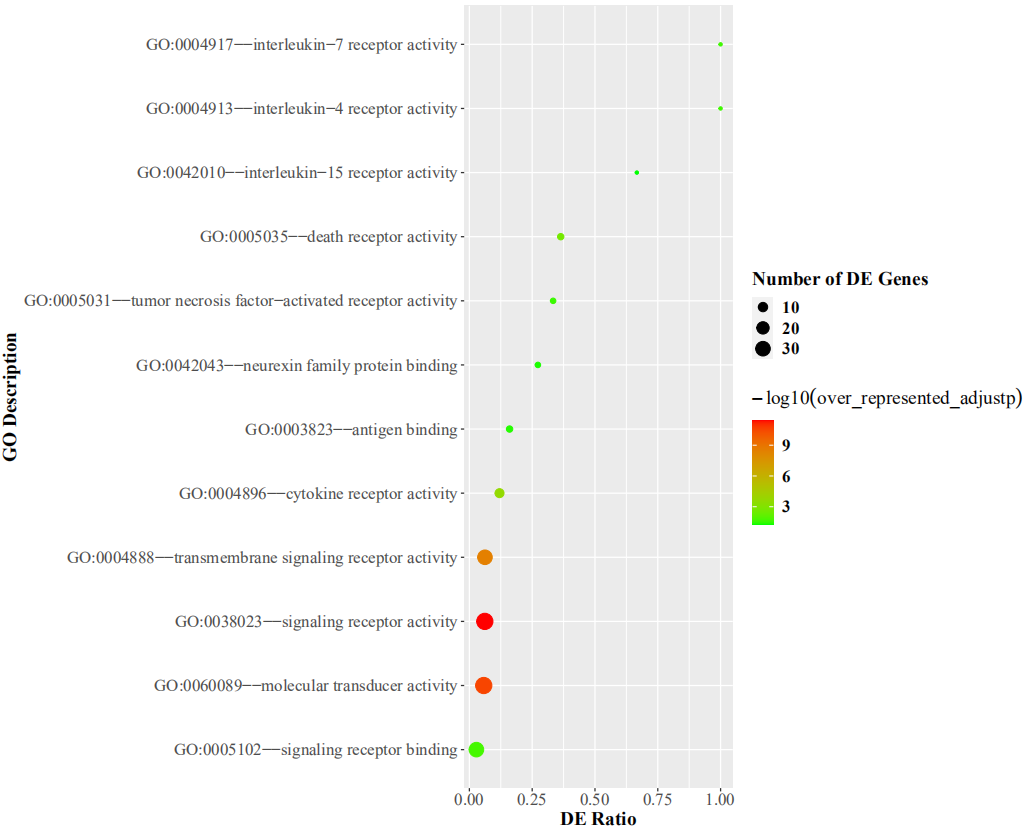


Figure S7: The dot plot of the top 20 gene ontologies in MF identified by GOseq package. The DE Ratio is the ratio of differentially expressed genes among all the genes in a specific GO category. The GO Description displays the ID and brief information of each GO. The color of dot shows the adjusted p value of the GO term. The size of dot represents the number of differentially expressed genes.


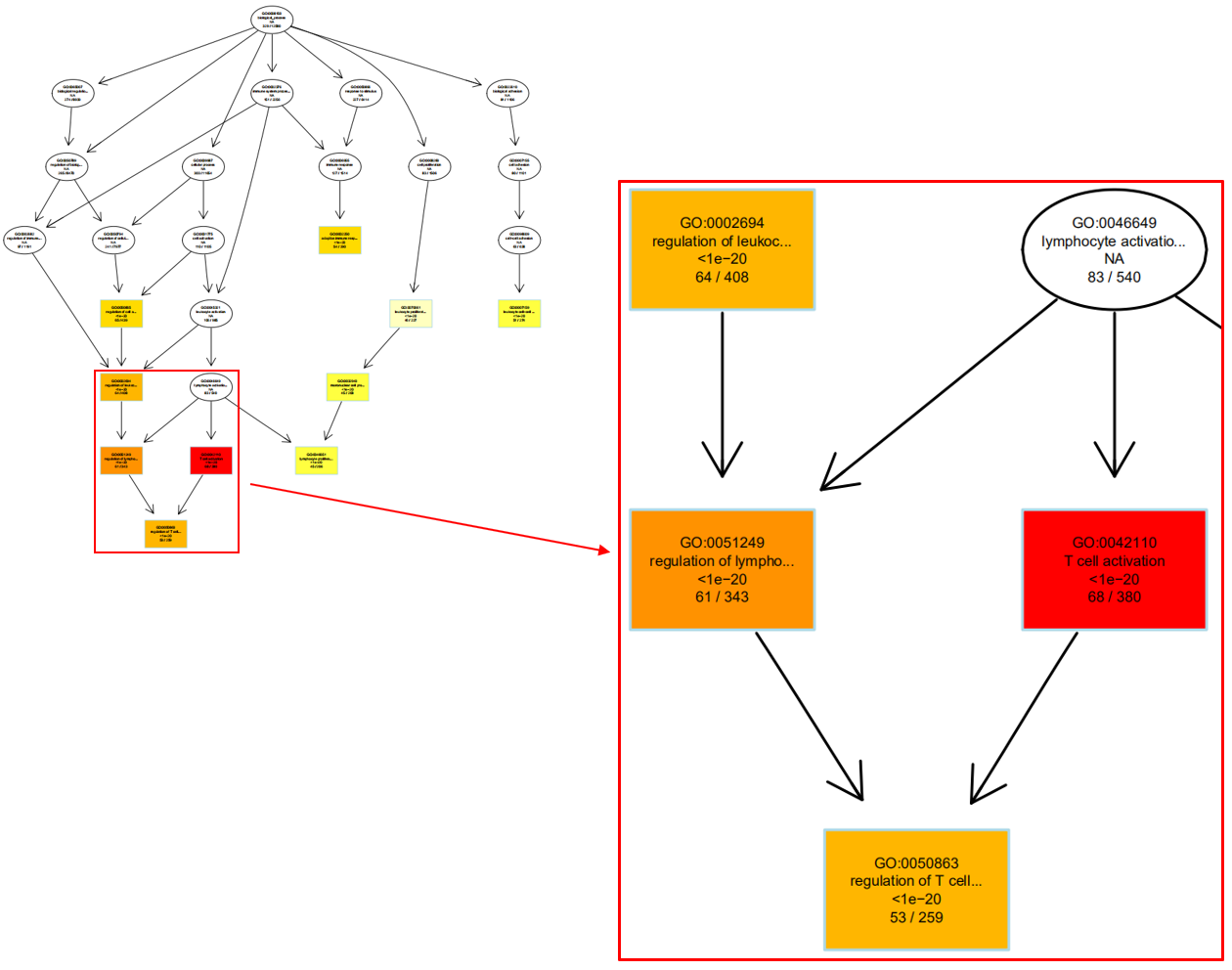


Figure S8: The directed acyclic graph of the enriched GO terms in biological process category identified by clusterProfiler package. The color represents the significance of GO terms (more significant from red to yellow). The arrow represents the hierarchical relationship between two terms. The shape of each term represents the top 10 significant GO terms (rectangle) and others (ellipse). In each term the GO ID, brief description, FDR, the number of differentially expressed genes and all genes were displayed.


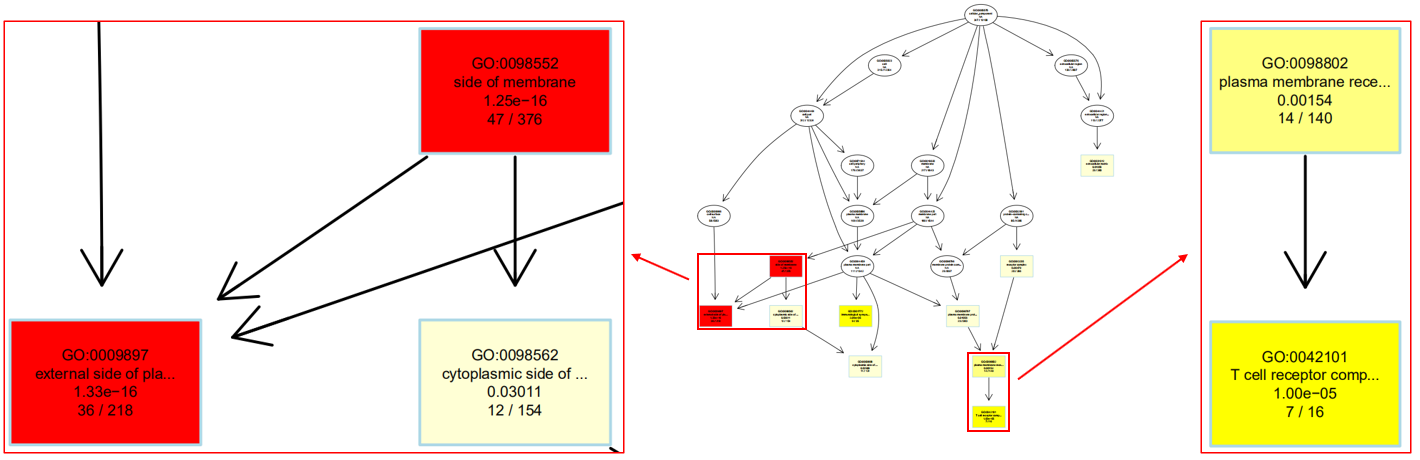


Figure S9: The directed acyclic graph of the enriched GO terms in cellular component category identified by clusterProfiler package. The color represents the significance of GO terms (more significant from yellow to red). The arrow represents the hierarchical relationship between two terms. The shape of each term represents the top 10 significant GO terms (rectangle) and others (ellipse). In each term the GO ID, brief description, FDR, the number of differentially expressed genes and all genes were displayed.


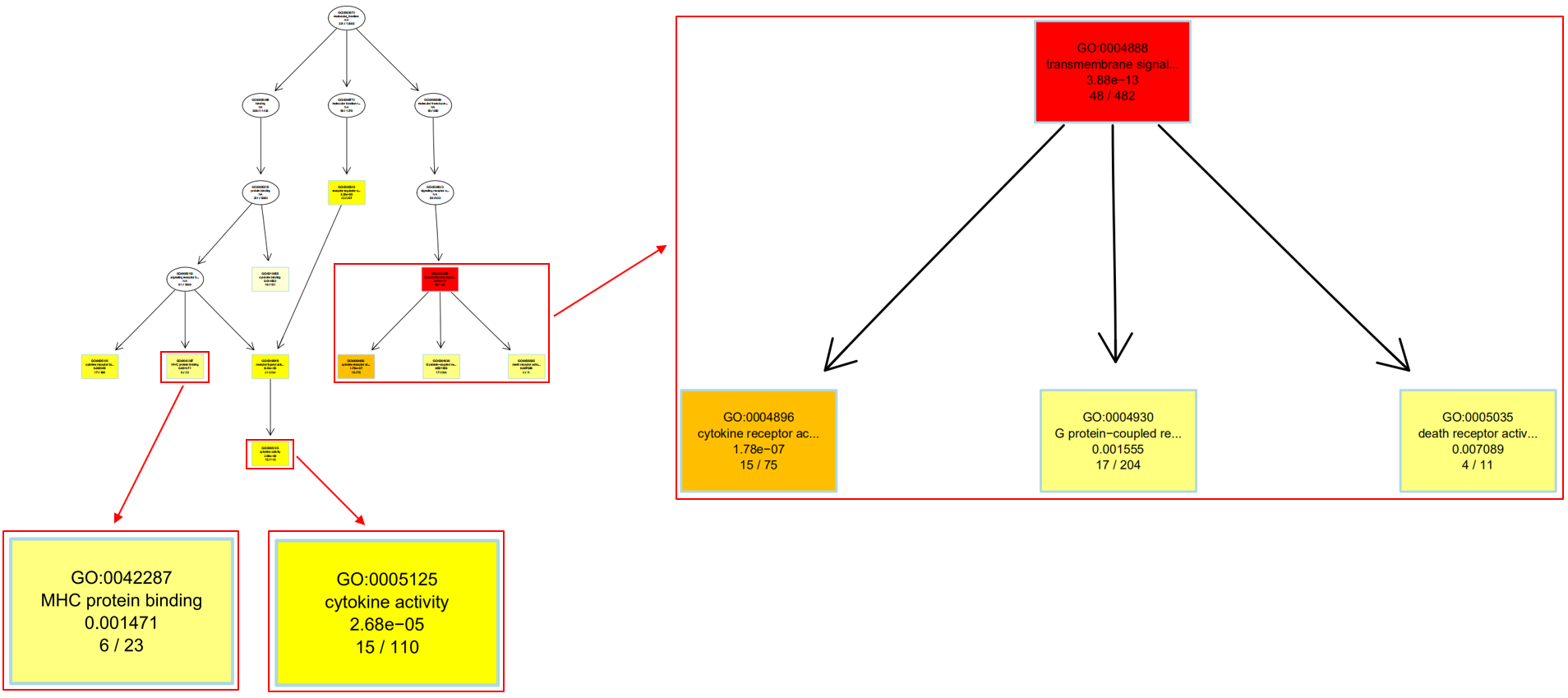


Figure S10: The directed acyclic graph of the enriched GO terms in molecular function category identified by clusterProfiler package. The color represents the significance of GO terms (more significant from yellow to red). The arrow represents the hierarchical relationship between two terms. The shape of each term represents the top 10 significant GO terms (rectangle) and others (ellipse). In each term the GO ID, brief description, FDR, the number of differentially expressed genes and all genes were displayed.

Figure S11: An illustration of WSI processing and feature extraction. A, image foreground segmentation; B, cropping global ROI; C, zooming in the selected global ROI; D, blocks sampling; E, nucleus segmentation; F, sampling nucleus and cropping its ROI; G, extracting texture features from each nucleus ROI; H, extracting texture features from global ROI.

# Supplementary Methods

***Whole slide image processing and feature extraction***

For region segmentation, we first applied OTSU(12) method to segment the foreground of WSI and retained the region with maximum area. We then segmented global and nucleus ROI from this region. For each 20X slide, the global ROI was defined as a block with a size of 16,000$\times$16,000 pixels, while the block size was 32,000$\times$32,000 for each 40X slide. We covered as much foreground as possible and finally the mean ratio of foreground areas in all global ROIs was 86%. To develop macro observation and speed up the computations, we shrunk all the global ROIs to 1000$\times$1000 pixels. For nucleus ROI, we first sampled 20 blocks with a size of 1000$\times$1000 pixels for 40X slides or 500$\times$500 pixels for 20X slides. These blocks were latter resized to 500$\times$500 pixels. We then segmented nuclei from these blocks by following steps:

1. Use color deconvolution(13) to convert each block from RGB channels into HEO channels;
2. Apply the locally adaptive threshold segmentation within each block to pre-identify the nuclei region and apply morphology opening to widen the segmentation edges(14);
3. Use OTSU to compute a global gray-level threshold in the H-channel image;
4. Retain pixels whose gray-level was higher than the threshold and who were located in the pre-identified nuclei regions;
5. Set area threshold to each connected component and perform morphology operations to optimize the shape.

The connected components were considered as the nuclei regions. We cropped the rectangle centering around a nucleus and included its 5-pixel dilation region as the nucleus ROI. For each block, only 20 nuclei were randomly sampled for further analysis. Three examples of nucleus segmentation were shown in Figure S1.

***Differential gene expression analysis***

For differential gene expression analysis, the TMM (the trimmed mean of M values) (21) method was used to normalize the expected gene count data. Only genes whose mean of counts was more than 15 reads and with at least 1 read in every sample were retained for normalization. This resulted in a total of 17,107 genes used for downstream analysis. The normalized counts were fitted into negative binomial GLM for differential expression analysis using edgeR(22) with tag-wise dispersion. Multiple testing was corrected by Benjamini-Hochberg procedure(23) to control the false discovery rate (FDR) and to obtain the adjusted p-values.

Gene ontology enrichment analysis was performed by GOseq(24), where the differentially expressed genes identified as described above were supplied as the input for genes of interest. The GOseq is able to adjust the confounding effects due to varying transcript lengths and expression levels when analyzing over-representation of gene categories, and is thus particularly suitable for RNA sequencing data. We also used clusterProfiler(25) as a comparison. The clusterProfiler is able to measure sematic similarity among GO terms to reduce the redundancy of GO enrichment results.

***Computational formulas of texture features included in the final model***

1. GLCM-based features:

$$ClusterShade=\sum_{i=1}^{N_{g}} \sum_{j=1}^{N_{g}} (i+j-\mu_{x}-\mu_{y})^{3}p(i,j)$$

$$DifferenceVariance=\sum_{k=0}^{N_{g}-1} (k-DA)^{2}p_{x-y}(k)$$

$$Idn=\sum_{k=0}^{N_{g}-1} \frac{p_{x-y}\left( k \right)}{1+\left( \frac{k}{N_{g}} \right)}$$

$$InverseVariance=\sum_{k=1}^{N_{g}-1} \frac{p_{x-y}(k)}{k^{2}}$$

$$Idmn=\sum_{k=0}^{N_{g}-1} \frac{p_{x-y}(k)}{1+(\frac{k^{2}}{{N_{g}}^{2}})}$$

$$MaximumProbability=max(p(i,j))$$

Notes:

$\mathbf{P}(i,j)$ is the co-occurrence matrix for an arbitrary$\delta$ and $\theta$;

$p(i,j)$ is the normalized co-occurrence matrix and equal to$\frac{P(i,j)}{\sum P(i,j)}$;

$N_{g}$ is the number of discrete intensity levels in the image;

$p_{x}(i)=\sum_{j=1}^{N_{g}} P(i,j)$ is the marginal row probabilities;

$p_{y}(j)=\sum_{i=1}^{N_{g}} P(i,j)$ is the marginal column probabilities;

$\mu_{x}$ is the mean gray level intensity of $p_{x}$ anddefined as $\mu_{x}=\sum_{i=1}^{N_{g}} p_{x}(i)i$;

$\mu_{y}$ is the mean gray level intensity of $p_{x}$ anddefined as $\mu_{y}=\sum_{j=1}^{N_{g}} p_{y}\left( i \right)i$

$p_{x+y}(k)=\sum_{i=1}^{N_{g}} \sigma_{j=1}^{N_{g}}p(i,j)$, where $i+j=k$, and $k=2,3,\ldots,2N_{g}$;

$p_{x-y}(k)=\sum_{i=1}^{N_{g}} \sigma_{j=1}^{N_{g}}p(i,j)$, where $|i-j|=k$, and $k=0,1,\ldots,N_{g}-1$.

1. GLRLM-based features:

$$GrayLevelNonUniformity=\frac{\sum_{i=1}^{N_{g}} \left( \sum_{j=1}^{Nr} P(i,j|\theta) \right)}{Nr(\theta)}$$

$$RunEntropy=-\sum_{i=1}^{N_{g}} \sum_{j=1}^{N_{r}} p(i,j|\theta) {log}_{2}(p(i,j|\theta)+\epsilon)$$

$$ShortRunEmphasis=\frac{\sum_{i=1}^{N_{g}} \sum_{j=1}^{N_{r}} \frac{p(i,j|\theta)}{j^{2}}}{Nr(\theta)}$$

$$ShortRunLowGrayLevelEmphasis=\frac{\sum_{i=1}^{N_{g}} \sum_{j=1}^{N_{r}} \frac{p(i,j|\theta)}{i^{2}j^{2}}}{Nr(\theta)}$$

Notes:

$N_{g}$ is the number of discreet intensity values in the image;

$N_{r}$ is the number of discreet run lengths in the image;

$N_{r}(\theta)$ is the number of runs in the image along angle $\theta$, which is equal to $\sum_{i=1}^{N_{g}} \sum_{j=1}^{N_{r}} \mathbf{P}(i,j|\theta)$ and $1\leq N_{r}(\theta)\leq N_{p}$;

$\mathbf{P}(i,j|\theta)$ is the run length matrix for an arbitrary direction $\theta$;

$p(i,j|\theta)$ is the normalized run length matrix, defined as $p(i,j|\theta)=\frac{P(i,j|\theta)}{N_{r}(\theta)}$.

1. GLSZM-based features:

$$LargeAreaHighGrayLevelEmphasis=\frac{\sum_{i=1}^{N_{g}} \sum_{j=1}^{N_{r}} P(i,j)i^{2}j^{2}}{N_{z}}$$

$$SizeZoneNonUniformityNormalized=\frac{\sum_{j=1}^{Ns} (\sum_{i=1}^{Ng} P(i,j))^{2}}{{N_{z}}^{2}}$$

$$GrayLevelNonUniformityNormalized=\frac{\sum_{i=1}^{N_{g}} (\sum_{j=1}^{N_{s}} P(i,j))^{2}}{N_{z}}$$

Notes:

$N_{g}$ is the number of discreet intensity values in the image;

$N_{s}$ is the number of discreet zone sizes in the image;

$N_{p}$ is the number of voxels in the image;

$N_{z}$ is the number of zones in the ROI, which is equal to $\sum_{i=1}^{N_{g}} \sum_{j=1}^{N_{s}} \mathbf{P}(i,j)$ and $1\leq N_{z}\leq N_{p}$;

$\mathbf{P}(i,j)$ is the size zone matrix;

$p(i,j)$ is the normalized size zone matrix, defined as $p(i,j)=\frac{\mathbf{P}(i,j)}{N_{z}}$.

**Reference**

12. N. O. A threshold selection method from gray-level histograms. IEEE transactions on systems, man, and cybernetics. 1979;9(1):62-6.

13. Ruifrok AC, Johnston DA. Quantification of histochemical staining by color deconvolution. Anal Quant Cytol Histol. 2001;23(4):291-9.

14. Lu C MM. Automated analysis and diagnosis of skin melanoma on whole slide histopathological images[J]. Pattern Recognition. Pattern Recognition. 2015;48(8):2738-50.

15. van Griethuysen JJM, Fedorov A, Parmar C, Hosny A, Aucoin N, Narayan V, et al. Computational Radiomics System to Decode the Radiographic Phenotype. Cancer Res. 2017;77(21):e104-e7.

16. Doyle S, Feldman MD, Shih N, Tomaszewski J, Madabhushi A. Cascaded discrimination of normal, abnormal, and confounder classes in histopathology: Gleason grading of prostate cancer. BMC Bioinformatics. 2012;13:282.

17. Jones S YZ, Xie Z, et al. A Proposed Data Analytics Workflow and Example Using the R Caret Package.

18. Tibshirani R. The lasso method for variable selection in the Cox model. Stat Med. 1997;16(4):385-95.

19. Witten DM, Tibshirani R. Survival analysis with high-dimensional covariates. Stat Methods Med Res. 2010;19(1):29-51.

20. Heagerty PJ, Lumley T, Pepe MS. Time-dependent ROC curves for censored survival data and a diagnostic marker. Biometrics. 2000;56(2):337-44.

21. Robinson MD, Oshlack A. A scaling normalization method for differential expression analysis of RNA-seq data. Genome Biol. 2010;11(3):R25.

22. Robinson MD, McCarthy DJ, Smyth GK. edgeR: a Bioconductor package for differential expression analysis of digital gene expression data. Bioinformatics. 2010;26(1):139-40.

23. Benjamini Y HY. Controlling the false discovery rate: a practical and powerful approach to multiple testing. Journal of the Royal statistical society: series B (Methodological). 1995;57(1):289-300.

24. Young MD, Wakefield MJ, Smyth GK, Oshlack A. Gene ontology analysis for RNA-seq: accounting for selection bias. Genome Biol. 2010;11(2):R14.

25. Yu G, Wang LG, Han Y, He QY. clusterProfiler: an R package for comparing biological themes among gene clusters. OMICS. 2012;16(5):284-7.

26. Pastorfide GC, Kibbi AG, de Roa AL, Barnhill RL, Sober AJ, Mihm MC, Jr., et al. Image analysis of stage 1 melanoma (1.00-2.50 mm): lymphocytic infiltrates related to metastasis and survival. J Cutan Pathol. 1992;19(5):390-7.

27. Kornstein MJ, Brooks JS, Elder DE. Immunoperoxidase localization of lymphocyte subsets in the host response to melanoma and nevi. Cancer Res. 1983;43(6):2749-53.

28. Ralfkiaer E, Hou-Jensen K, Gatter KC, Drzewiecki KT, Mason DY. Immunohistological analysis of the lymphoid infiltrate in cutaneous malignant melanomas. Virchows Arch A Pathol Anat Histopathol. 1987;410(4):355-61.
